# Dosimetric comparison of FF and FFF modes under non‐ideal bolus‐fitting conditions for post‐mastectomy chest wall irradiations

**DOI:** 10.1002/acm2.70653

**Published:** 2026-06-08

**Authors:** Long‐Gang Gui, Yin Poo

**Affiliations:** ^1^ School of Electronic Science and Engineering Nanjing University Nanjing China; ^2^ Radiotherapy Center Northern Jiangsu People's Hospital Yangzhou Yangzhou China

**Keywords:** bolus fit, chest wall radiotherapy, curved phantom, dose heterogeneity index, Monte Carlo algorithm

## Abstract

**Background:**

In post‐mastectomy radiotherapy, tissue‐equivalent bolus is routinely used to ensure adequate skin dose. However, achieving perfect contact on curved chest walls is challenging, often resulting in non‐uniform air gaps. With the increasing adoption of Flattening Filter‐Free (FFF) beams, it is crucial to understand how their distinct physical properties—specifically a softer energy spectrum—respond to these low‐density gaps compared to Flattening Filter (FF) beams. Additionally, the accuracy of clinical dose calculation algorithms in these electronic disequilibrium regions remains a subject of concern.

**Purpose:**

This study aimed to quantify the impact of non‐uniform air gaps on skin dose distribution, evaluate the accuracy of Collapsed Cone Convolution (CCC) versus GPU‐accelerated Monte Carlo (MC) algorithms in complex geometries, and determine the specific dosimetric thresholds for selecting FF or FFF beam modes.

**Methods:**

A cylindrical curved phantom (25 cm diameter) was constructed to simulate chest wall anatomy. Four interchangeable modules were designed to create controlled fit conditions: perfect fit, gradient gap (0–6 mm), and uniform gaps (3 and 6 mm). Skin dose measurements were performed using EBT3 films and MOSFET detectors. Dose calculations were conducted in the United Imaging uRT‐TPS using 6 MV FF and FFF beams at gantry angles of 0°, 30°, and 60°.

**Results:**

The presence of gradient gaps substantially increased skin dose inhomogeneity, yielding a heterogeneity index of 0.28 for the FFF beam. The MC algorithm demonstrated high accuracy, agreeing with film measurements within 0.5%. In contrast, the CCC algorithm exhibited systematic underestimation in gap regions, with a mean error of −6.8% and a maximum deviation of −16.5%. Physically, the FFF beam (mean energy 1.23 MeV) was found to be more sensitive to air gaps than the FF beam (1.93 MeV) due to reduced lateral electron scatter. The FFF/FF dose ratio decreased linearly with increasing gap thickness (slope: −2.1%/mm). A clinical threshold was identified at approximately 2.3 mm; beyond this gap thickness, the FF beam exhibited superior dosimetric robustness. Furthermore, oblique incidence at 60° reduced the mean skin dose by approximately 9% compared to perpendicular incidence.

**Conclusion:**

Non‐uniform air gaps significantly degrade skin dose uniformity, with FFF beams being more susceptible to this effect than FF beams. The CCC algorithm shows limitations in these complex interface geometries, whereas the MC algorithm provides a reliable verification tool. Clinically, for regions where air gaps exceed 2.3 mm, the use of FF mode or optimization of bolus fitting is recommended to ensure adequate target coverage.

## 1 INTRODUCTION

Chest wall irradiation after modified radical mastectomy is a common clinical scenario in superficial tumor radiotherapy. To overcome the skin‐sparing effect of megavoltage (MV) photon beams and ensure adequate dose delivery to the skin and subcutaneous tissues, tissue‐equivalent bolus is routinely placed on the chest wall surface.^1^, ^2^, ^3^ However, achieving perfect contact with zero air gap is extremely difficult in clinical practice. Although three‐dimensional (3D) printed customized bolus has improved conformality in recent years,^4^, ^5^ conventional sheet bolus remains the mainstream choice in most radiotherapy centers due to the high cost and time‐consuming nature of customized fabrication. Given the curved anatomy of the chest wall, material stiffness, and gravitational effects, air gaps between the bolus and skin are often unavoidable. These gaps are typically smallest at the apex of the curved surface and gradually increase toward the periphery, exhibiting a complex non‐uniform gradient distribution (0–10 mm). From a physical perspective, the presence of air gaps does not simply cause photon attenuation; more importantly, it disrupts charged particle equilibrium (CPE).^6^ The low‐density air layer cannot generate sufficient secondary electrons to maintain the build‐up effect and leads to lateral electron scatter loss from the superficial deposition region, creating unpredictable dose cold spots in the subcutaneous layer.^7^ Such dose variations may directly compromise the clinical efficacy and safety of the treatment.

Currently, the collapsed cone convolution (CCC) algorithm, widely used in commercial treatment planning systems (TPSs), incorporates tissue heterogeneity corrections but has inherent limitations in handling thin tissue‐air interfaces and electron disequilibrium scenarios.^8^ In contrast, the Monte Carlo (MC) algorithm is recognized as the gold standard for dose calculation due to its ability to simulate electron transport more accurately.^9^, ^10^ Furthermore, with the increasing use of high‐dose‐rate flattening filter‐free (FFF) mode in chest wall radiotherapy, whether its softer energy spectrum (lower mean energy) leads to greater sensitivity to air gaps due to reduced secondary electron range has not been systematically quantified.

Previous studies have mostly focused on planar geometry or uniform air gaps of single thickness,^11^, ^12^ lacking systematic evaluation of the more clinically relevant gradient gaps. Few studies have simultaneously addressed algorithm validation and comparison of different beam modes (flattening filter [FF] vs. FFF). Therefore, in this study, we designed a cylindrical curved phantom with controllable gradient gaps to simulate realistic chest wall anatomy. By comparing EBT3 film measurements with CCC and GPU‐accelerated MC calculations in a commercial TPS, this study aimed to address the following clinical questions: (1) quantify the specific effects of non‐uniform gaps on skin dose distribution; (2) identify the failure boundary of the CCC algorithm under gradient gap conditions; and (3) investigate the dosimetric differences between FF and FFF modes under various gap conditions, thereby providing quantitative evidence for clinical bolus management and beam mode selection.

## 2 MATERIALS AND METHODS

### 2.1 Curved phantom and fit module design

**FIGURE 1:**
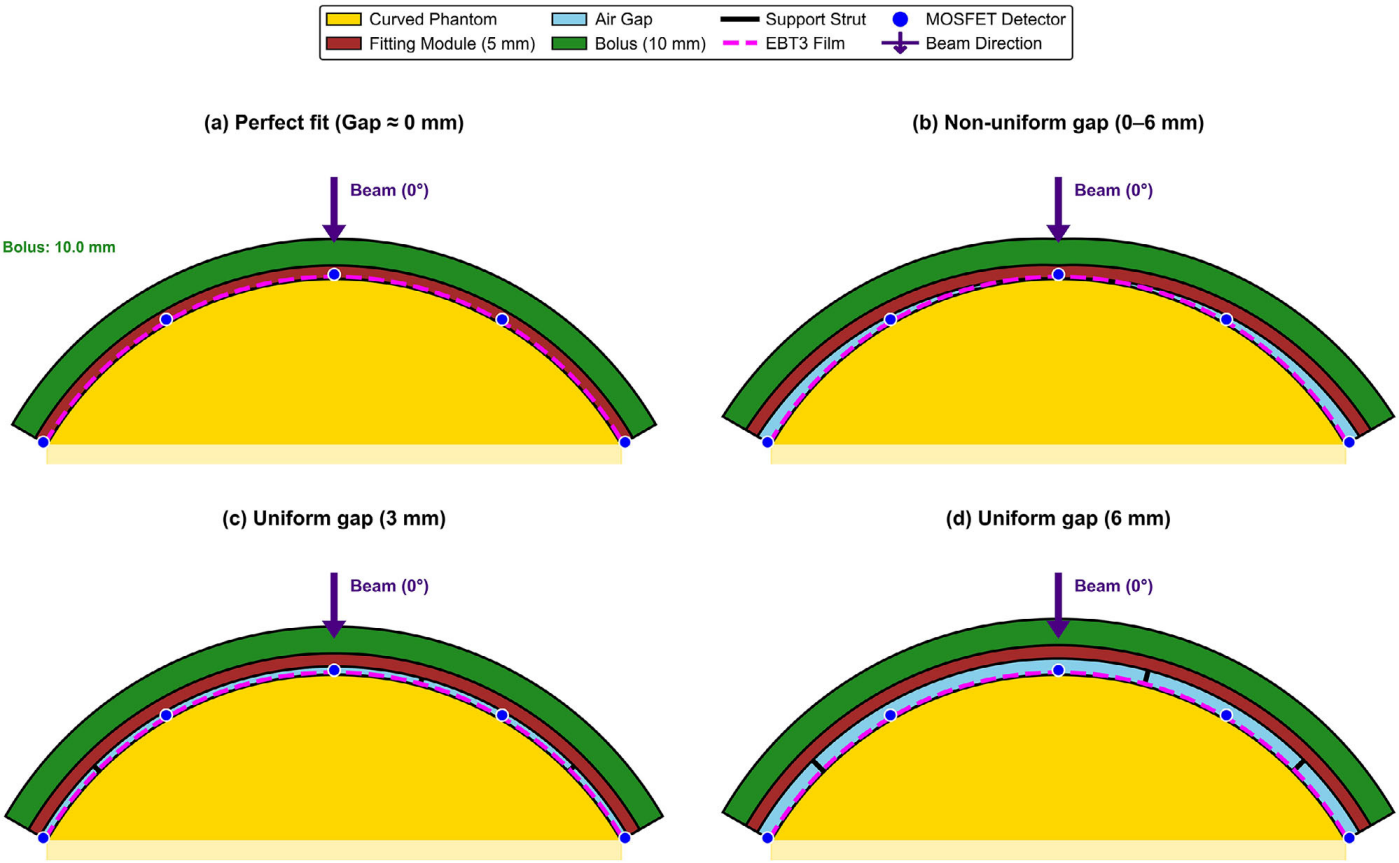
Schematic diagram of the curved anatomical phantom design and the four bolus fit conditions.

A cylindrical polylactic acid (PLA) phantom with a diameter of 25 cm was constructed to simulate the average anatomical curvature of the adult central chest wall. As illustrated in Figure 1, four interchangeable bolus‐fitting modules (each 5 mm thick) were fabricated to create controlled air gaps: the ideal‐fit module (Type‐A) had an inner surface perfectly matching the phantom curvature, with a CT‐verified actual gap of < 0.5 mm, simulating an ideal clinical fit; the gradient gap module (Type‐B) simulated non‐uniform separation caused by gravity, with the air gap increasing linearly from 0 mm at the apex to 6 mm at the bilateral edges; the uniform gap modules (Type‐C and Type‐D) utilized three miniature support struts (3 mm in diameter) to generate uniform air layers of 3 and 6 mm, respectively, serving as controls to isolate the dosimetric effect of gap thickness. All modules featured uniformly curved outer surfaces to concentrically accommodate a 10‐mm‐thick tissue‐equivalent bolus. The entire experimental measurement setup was manufactured from acrylonitrile butadiene styrene (ABS) material (density = 1.04 g/cm^3^), primarily chosen for its excellent dosimetric tissue equivalence and structural stability, which have been well validated in clinical radiotherapy applications.^12^

### 2.2 Dose measurement system and film processing

Gafchromic EBT3 film (Ashland Inc., NJ, USA) was used for two‐dimensional (2D) skin dose measurements following the AAPM TG‐55 and TG‐235 recommendations for radiochromic film dosimetry. Films were placed tightly on the phantom surface (at the 12.5 cm radius curvature). All films were stored for 24 hours after irradiation to allow stabilization of the polymerization process.

Digital scanning was performed using an Epson Expression 10 000XL flatbed scanner in transmission mode (48‐bit RGB, 72 dpi) with all image enhancement features disabled. The red channel was extracted for optical density‐to‐dose conversion due to its highest sensitivity in the low‐dose region (< 10 Gy). Triple‐channel non‐uniformity correction was applied to eliminate scanner lamp non‐uniformity and lateral effects. Dose calibration curves were established in solid water at the depth of maximum dose (d_max_ = 1.5 cm, source‐to‐surface distance [SSD] = 100 cm) for a 6 MV beam over a dose range of 0–500 cGy, fitted with a third‐order polynomial (*R* ^2^ > 0.99). Considering film uniformity, scanner stability, and fitting errors, the overall uncertainty of film dosimetry was estimated to be approximately 2.5% (*k* = 1).

Point dose verification was performed using Standard Imaging A18S MOSFET detectors operated in high‐sensitivity mode, placed at specific angles on the phantom surface (0°, ± 30°, ± 60°). Detectors were pre‐calibrated in a known dose field and corrected for angular response.

### 2.3 Treatment planning and algorithm settings

**FIGURE 2:**
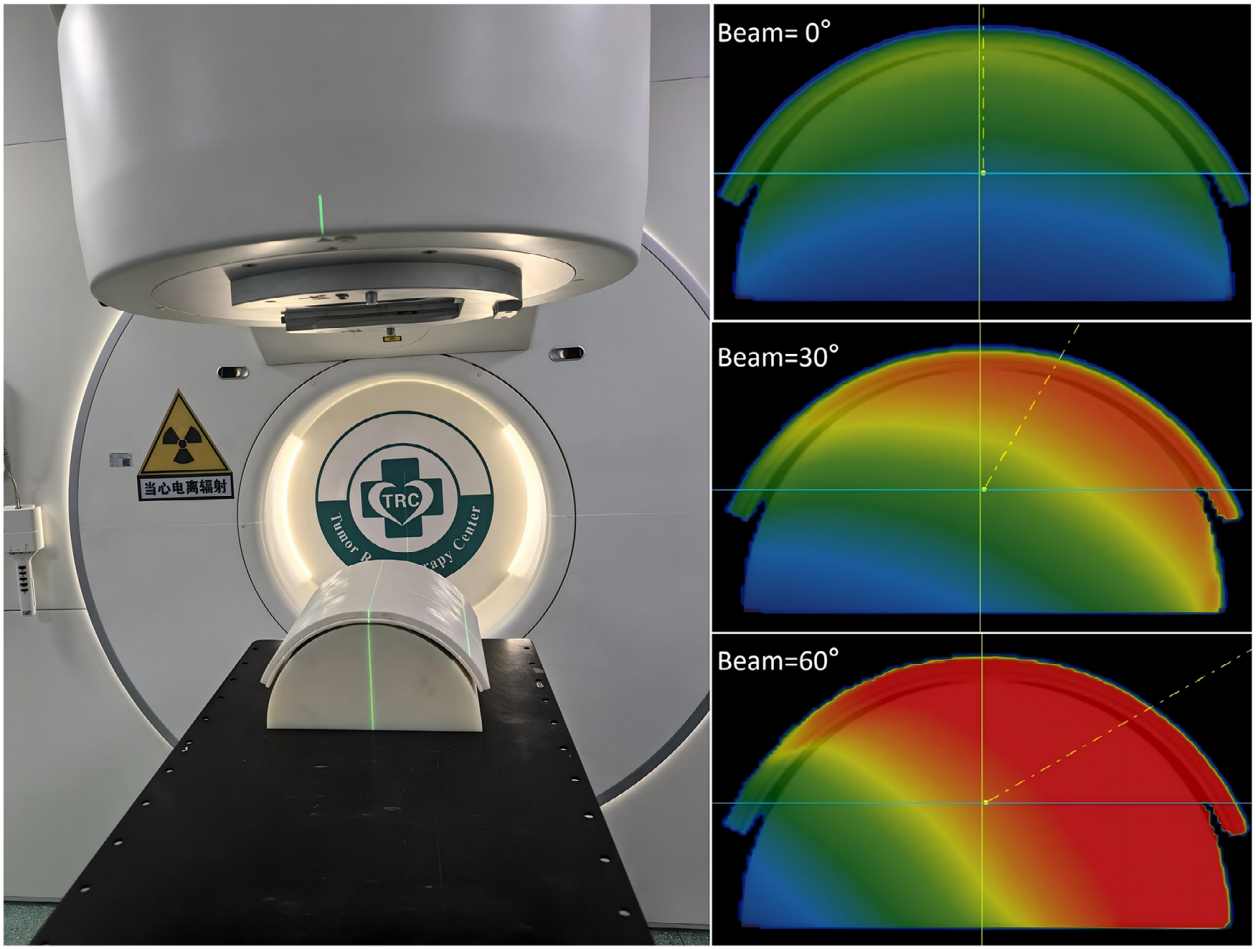
The actual physical measurement setup (left) and the corresponding dose calculation screenshots in the uRT‐TPS (right).

The uRT‐linac 306 (United Imaging Healthcare, Shanghai, China) is a modern integrated linear accelerator equipped with a 120‐leaf multi‐leaf collimator (MLC). Its commercial treatment planning system (uRT‐TPS) integrates both CCC and MC dose calculation algorithms. In particular, the GPU‐accelerated MC engine utilizes highly parallelized computation to significantly improve dose calculation speed in heterogeneous media. The actual physical measurement setup and the corresponding dose calculation screenshots in the uRT‐TPS are shown in Figure 2.

The phantom assembled with different fit modules was scanned using computed tomography (CT) with a slice thickness of 1.25 mm, and the images were imported into a commercial treatment planning system (uRT‐TPS, United Imaging Healthcare). A 5‐mm thick layer beneath the surface was contoured as the skin evaluation volume. Treatment plans were generated using a uRT‐linac 306 linear accelerator model configured for both 6 MV flattened (FF) and FFF photon beams. To isolate the physical effects of interest and eliminate interference from MLC modulation, a single open field of 22 × 22 cm^2^ was employed. To independently evaluate the effect of beam incidence, three separate treatment plans were generated for each fit module with gantry angles individually set to 0° (perpendicular incidence), 30°, and 60° (oblique incidence). Two dose calculation algorithms were compared: the CCC algorithm (calculation grid: 2 mm; standard tissue heterogeneity correction enabled) and the GPU‐accelerated MC algorithm built into the uRT‐TPS (reporting Dose‐to‐Medium,D_m_). To ensure high calculation accuracy for the MC algorithm, the calculation grid was refined to 1 mm, and the statistical uncertainty within the Region of Interest (ROI) was set to 1% per voxel. All plans were normalized such that the dose at the geometric center of the phantom (depth: 6.25 cm) was 1000 cGy, facilitating direct relative dose comparisons across different experimental conditions.

### 2.4 Statistical analysis and evaluation metrics

Statistical analysis was performed using SPSS version 26.0. A three‐way analysis of variance (ANOVA) was conducted on 48 plan sets (4 modules × 2 beam modes × 2 algorithms × 3 angles, with 5 repeated measurements per set) to examine main effects and interactions of algorithm, beam mode, and incidence angle, with a significance level of α = 0.05. The dose heterogeneity index (HII) was defined as:

$$\mathrm{HII}=\frac{D_{2\%}-D_{98\%}}{D_{50\%}}$$

where *D*_2%_, *D*_98%_, and *D*_50%_ represent the near‐maximum, near‐minimum, and median doses received by the skin layer volume, respectively. HII values closer to 0 indicate more uniform dose distribution, while larger values indicate greater inhomogeneity. To assess the magnitude of dose reduction at the peripheral curved geometry due to air gaps, the side‐to‐top ratio (STR) was defined as:

$$\mathrm{STR}=\frac{D(\theta =60^{\circ })}{D(\theta =0^{\circ })}\;\times 100\%$$

Algorithm accuracy was evaluated by relative error (%Δ*D*), using EBT3 film measurements (*D*_meas_) as the reference standard:

$$\%\Delta D=\frac{D_{\mathrm{calc}}-D_{\mathrm{meas}}}{D_{\mathrm{meas}}}\times 100\%$$

where *D*_calc_ represents the dose calculated by the TPS (CCC or MC). Statistical differences were compared using paired sample *t*‐tests, with a significance level of *P* < 0.05.

## 3 RESULTS

### 3.1 Dosimetric effects of non‐uniform gaps under perpendicular incidence

**FIGURE 3:**
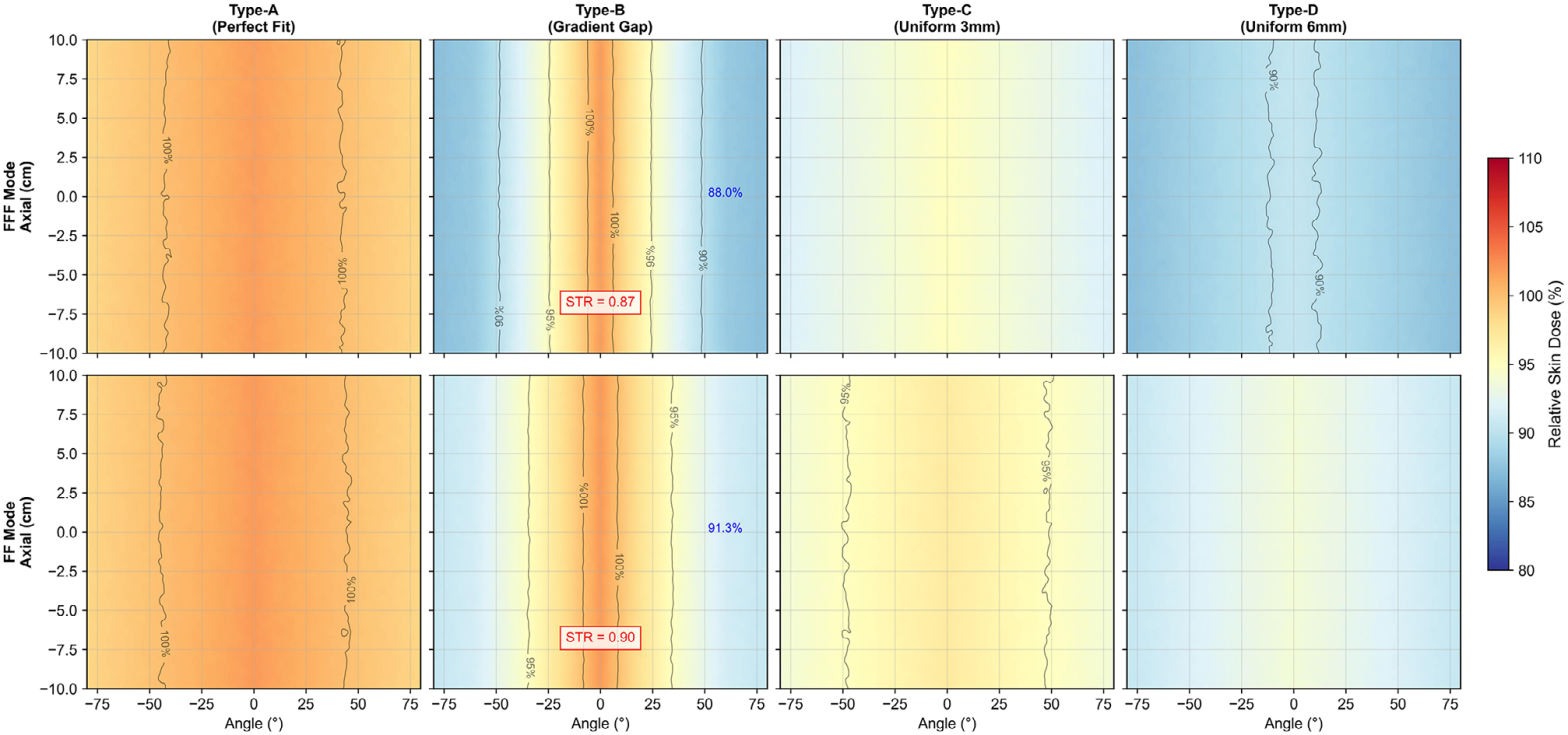
2‐D skin dose distributions for different bolus fit conditions and beam modes (FF/FFF) under perpendicular incidence.

Table 1 and Figure 3 present the skin dose distribution characteristics for the four fit conditions under FF and FFF beam modes. Under the ideal perfect fit condition (Type‐A), both beam modes exhibited good dose uniformity, with STR values near 99.0% and HII values below 0.05, indicating adequate CPE. However, the introduction of the gradient gap (Type‐B) resulted in a clear dose reduction as the gap thickness increased. Specifically, at lateral positions where the gap reached 6 mm (± 60°), the relative skin dose decreased to 84.5% ± 2.1% for the FFF mode, compared to 89.2% ± 1.8% for the FF mode. Correspondingly, the STR for the FFF mode dropped to 83.1% ± 1.9%, which was significantly lower than the 87.3% ± 1.9% observed for the FF mode (*P* < 0.01). This indicates that the FFF mode produces a steeper dose gradient under non‐uniform gap conditions.

**TABLE 1:** STR and HII for different fit conditions under perpendicular incidence.

Fit Type	Gap characteristics	FFF‐STR (%)	FF‐STR (%)	FFF‐HII	FF‐HII
Type‐A	Perfect fit (< 0.5 mm)	98.8 ± 1.5	99.1 ± 1.3	0.04 ± 0.02	0.03 ± 0.02
Type‐B	Gradient gap (0–6 mm)	83.1 ± 1.9	87.3 ± 1.9	0.28 ± 0.05	0.22 ± 0.04
Type‐C	Uniform 3 mm gap	96.1 ± 1.7	96.9 ± 1.5	0.09 ± 0.03	0.08 ± 0.03
Type‐D	Uniform 6 mm gap	92.2 ± 1.9	94.1 ± 1.8	0.15 ± 0.04	0.13 ± 0.04

This difference is primarily due to the distinct energy spectra of the two beam modes. Due to the removal of the FF, the FFF beam has a softer photon energy spectrum. This produces secondary electrons with lower mean energy and shorter ranges, making the FFF mode more sensitive to the loss of lateral electron scatter caused by air gaps.^13^, ^14^

Notably, although uniform gaps (Type‐C/D) reduced the overall dose level (mean dose approximately 90% for the 6‐mm gap), their HII values (0.13–0.15) were consistently lower than those of the gradient gap group (0.22–0.28). This suggests that the gradient variation of the air gap, rather than the gap thickness alone, is the primary driver of severe local dose inhomogeneity, causing simultaneous overexposure at contact points and underdosing in gap regions.

### 3.2 Accuracy of dose calculation algorithms in non‐uniform gaps

**FIGURE 4:**
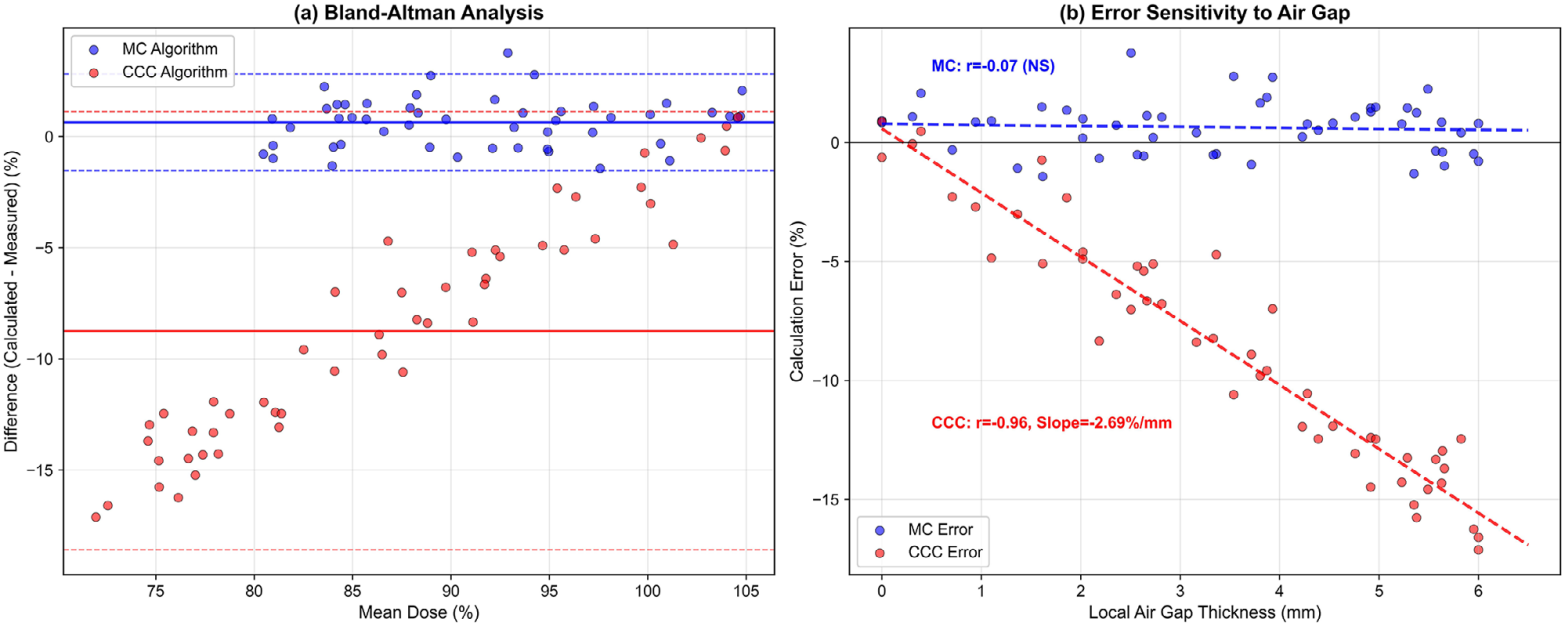
Evaluation of calculation accuracy for MC and CCC algorithms in curved geometry with air gaps.

Table 2 and Figure 4 provide a detailed comparison of calculation accuracy between the MC algorithm and the CCC algorithm under different fit conditions. Under the ideal condition of perfect fit (Type‐A), both algorithms demonstrated excellent accuracy, with mean relative errors within ± 0.5% and maximum deviations not exceeding 2.2%. However, with the introduction of air gaps, the calculation performance of the CCC algorithm decreased substantially. For the gradient gap (Type‐B), the CCC algorithm showed systematic underestimation, with a mean error of −6.8% ± 4.5% and a maximum negative deviation of −16.5% (occurring at the location of the largest gap). In contrast, the MC algorithm remained highly stable across all test conditions, with a mean error of only 0.5% ± 1.2%, and the majority of data points fell within the ± 3% limits of agreement (95% LoA, Figure 4a).

**TABLE 2:** Accuracy of dose calculation algorithms (MC vs. CCC) under different fit conditions.

Fitting type	Gap condition	MC mean error (%Δ*D*)	MC max deviation	CCC mean error (%Δ*D*)	CCC max deviation
Type‐A	Perfect Fit (< 0.5 mm)	0.3 ± 0.8	1.5	−0.5 ± 1.1	−2.2
Type‐B	Gradient gap (0–6 mm)	0.5 ± 1.2	−2.8	−6.8 ± 4.5	−16.5
Type‐C	Uniform 3 mm	0.4 ± 1.0	−1.9	−3.2 ± 1.8	−5.4
Type‐D	Uniform 6 mm	−0.2 ± 1.1	−2.5	−5.8 ± 2.2	−8.9

Further correlation analysis (Figure 4b) revealed the source of this error. The calculation error of the CCC algorithm showed a strong negative correlation with local air gap thickness (*r* = −0.92, *P* < 0.001), with prediction bias increasing approximately linearly as the gap increased from 0 to 6 mm (slope approximately −2.5%/mm). Even under uniform gap conditions (Type‐C/D), the CCC algorithm still exhibited notable underestimation (mean error of −5.8% for Type‐D), although this was somewhat moderated compared to its extreme deviations under gradient gaps. These findings confirm that the CCC algorithm has inherent modeling limitations when handling electron lateral scatter in low‐density media and complex interfaces, while the MC algorithm can accurately simulate electron transport under non‐equilibrium conditions and serves as the preferred tool for dose verification in complex fit scenarios.

### 3.3 Effect of beam incidence angle on skin dose

**FIGURE 5:**
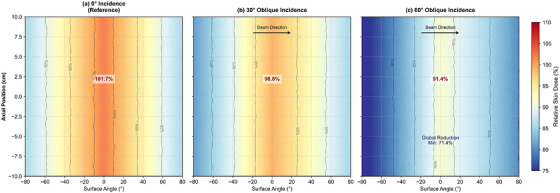
Effect of beam incident angle on skin dose distribution under non‐uniform gap (Type‐B) condition (FFF mode).

Table 3 and Figure 5 illustrate the impact of beam incidence angle (0°, 30°, and 60°) on skin dose distribution. As the incidence angle increased, the overall skin dose level showed a marked decreasing trend. Under perpendicular incidence (0°), the mean skin dose was 95.2% ± 3.5%, but this value decreased to 86.5% ± 5.2% under 60° oblique incidence (*P* < 0.01). This dose reduction was particularly evident in the gradient gap model (Type‐B): due to the obliquity effect, which shifts the depth of electron build‐up deeper as the incidence angle increases, combined with the air gap blocking the replenishment of backscattered electrons, the minimum skin dose (cold spot) dropped sharply from 84.5% to 74.8%.

**TABLE 3:** Effect of different incident angles on skin dose distribution parameters (Type‐B, FFF mode).

Incident angle (°)	Mean skin dose (%)	Minimum dose (cold Spot, %)	HII
0° (Perpendicular)	95.2 ± 3.5	84.5	0.28 ± 0.05
30° (Oblique)	91.8 ± 4.1	80.2	0.32 ± 0.06
60° (Oblique)	86.5 ± 5.2	74.8	0.38 ± 0.07

In addition, oblique incidence altered the spatial symmetry of the dose distribution. As the angle increased, a distinct downstream shift was observed, with the high‐dose region moving along the beam direction. Meanwhile, the upstream region experienced more extensive low‐dose areas due to severe loss of lateral electron scatter. This resulted in the dose heterogeneity index (HII) worsening from 0.28 at 0° to 0.38 at 60°. These findings suggest that when treating chest wall with tangential fields in the presence of substantial air gaps, large‐angle oblique incidence can lead to significant underdosing of the skin, and the actual delivered dose may be considerably lower than predicted by the TPS, even if the plan appears acceptable.

### 3.4 Influence of beam filtration mode

**FIGURE 6:**
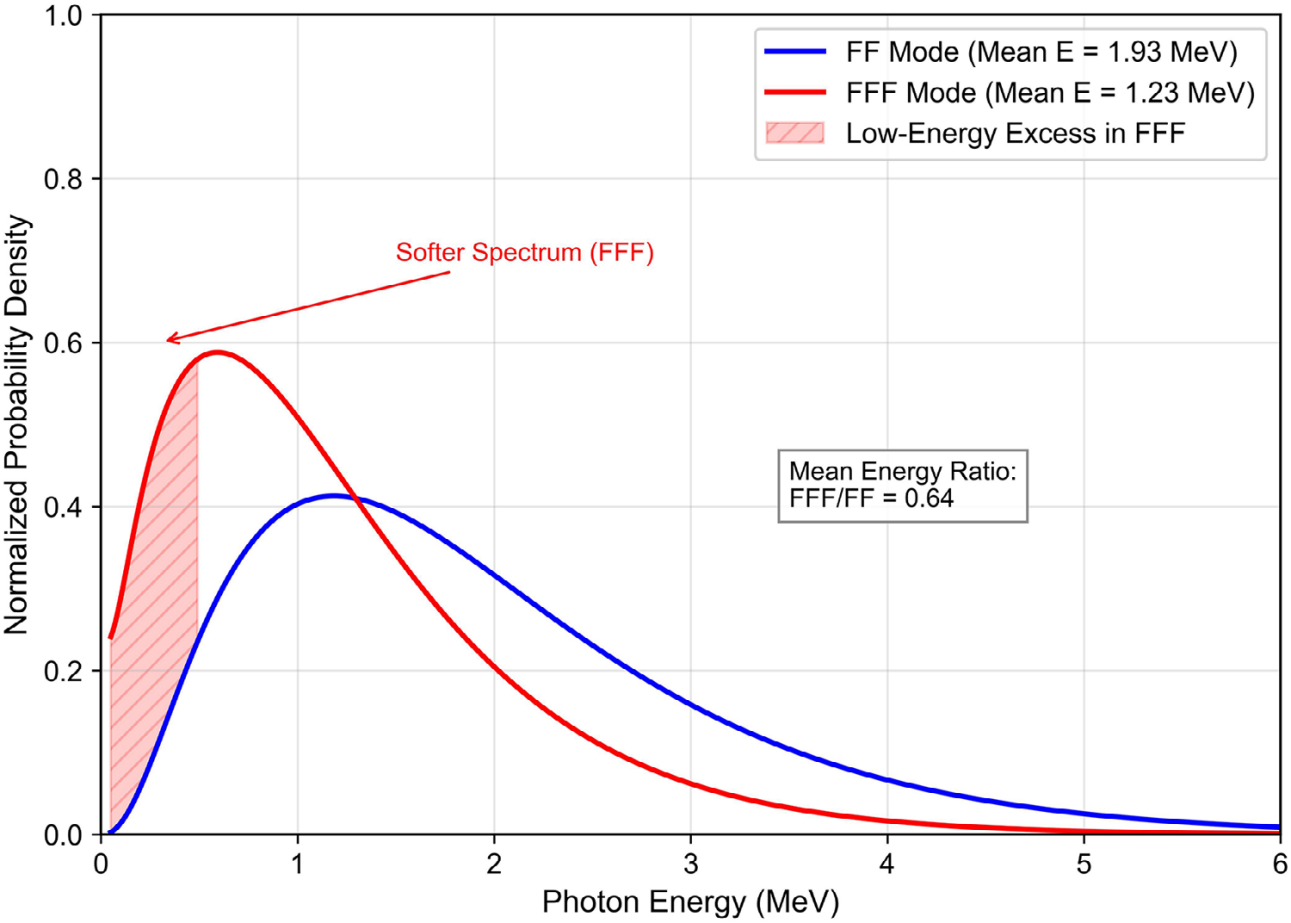
Energy spectrum characteristics of 6 MV FF and FFF photon beams.

To better understand the differential dose response of the two beam modes under non‐uniform gaps, the physical energy spectra of FF and FFF modes were first analyzed (Figure 6). Due to the removal of the FF, the FFF beam spectrum did not undergo the hardening process and therefore retained a substantial proportion of low‐energy photons (< 0.5 MeV). Calculations showed that the mean photon energy of the FFF mode was approximately 1.23 MeV, considerably lower than that of the FF mode at 1.93 MeV. This softer spectrum directly results in secondary electrons with lower mean energy and shorter range. While this characteristic provides higher surface dose in tissue‐equivalent media, it also makes the FFF mode more sensitive to variations in medium density, such as the introduction of air gaps.^15^

**FIGURE 7:**
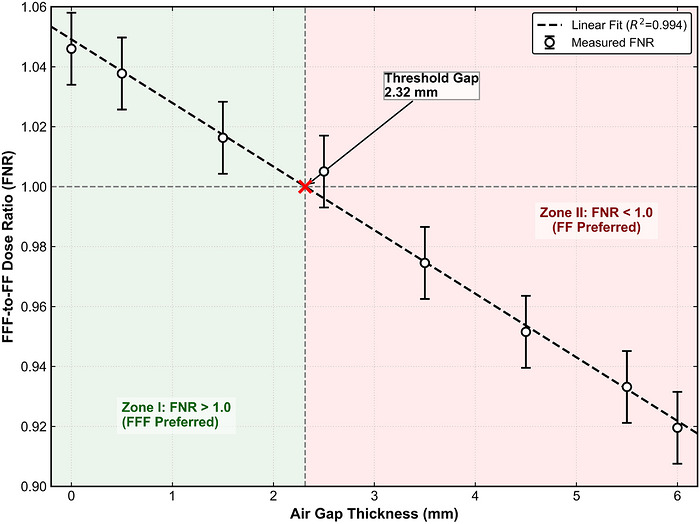
FFF/FF dose ratio (FNR) as a function of air gap thickness.

This physical difference was quantitatively verified by the trend of the dose ratio (FNR) as a function of gap thickness (Figure 7). In regions with small gaps where fit was relatively tight (< 1.0 mm), the FFF mode demonstrated superior surface dose coverage due to its abundance of low‐energy components, with FNR values maintained above 1.0 (approximately 1.045). However, as the air gap increased, the dose reduction rate for the FFF mode was notably faster than that for the FF mode, manifested as a linear decrease in FNR (slope approximately −0.021/mm, *R* ^2^ > 0.98). This indicates that air gaps cause considerably greater loss of lateral electron scatter for the FFF mode than for the FF mode.

Based on linear regression analysis, the clinical threshold where FNR = 1.0 was determined. The analysis showed that this threshold occurred at a gap thickness of approximately 2.3 mm. When the local gap was smaller than this threshold, the FFF mode was able to deliver higher skin dose; once the gap exceeded 2.3 mm, the FF mode demonstrated better robustness in maintaining surface dose due to its harder spectrum and greater electron penetration capability (FNR < 1.0). This quantitative threshold provides a direct physical basis for beam mode selection in clinical situations with complex fit conditions, suggesting that for sites where tight fit cannot be ensured (such as areas with irregular scarring), priority consideration of the FF mode may be more appropriate.

A comprehensive summary of the physical characteristics and the corresponding dosimetric differences between the FF and FFF modes is presented in Table 4.

**TABLE 4:** Comparison of physical characteristics and dosimetric properties between FF and FFF beam modes.

Characteristic parameter	FF mode	FFF mode	FNR trend / comparison
Mean photon energy	1.93 MeV	1.23 MeV	FFF softer (∼64% of FF)
Low‐energy fraction (< 0.5 MeV)	∼12.5%	∼28.4%	Significant excess in FFF
Surface dose ratio (Gap ≈ 0 mm)	Reference (1.0)	1.045	FFF advantage (Zone I)
Surface dose ratio (Gap = 6 mm)	Reference (1.0)	0.918	FF advantage (Zone II)
FNR decay slope	–	–	−2.1% per mm
Clinical threshold (FNR = 1.0)	–	–	Gap thickness ≈ 2.32 mm

*Note*: “–” indicates parameters that are common characteristics derived from the comparison between the two modes rather than belonging to a single mode.

## 4 DISCUSSION

This study, through MC simulations, investigated the dosimetric response differences of 6 MV photon beams in FFF and FF modes to non‐uniform air gaps. The core finding is that although the FFF mode provides higher surface dose under ideal fit conditions, it exhibits significantly greater sensitivity to air gaps compared to the FF mode. The physical origin of this difference lies primarily in the alteration of the energy spectrum following removal of the FF. As shown in our spectral analysis, the FFF mode retains a substantial number of photons with energies below 0.5 MeV, with a mean energy of 1.23 MeV, approximately 64% of that of the FF mode (1.93 MeV). According to Compton scattering principles, lower‐energy photons produce secondary electrons with shorter mean range and larger lateral scattering angles.^16^, ^17^ In tissue‐equivalent media, this characteristic contributes to enhanced surface dose; however, when a low‐density air gap is introduced, these low‐energy electrons are more prone to lateral escape or are blocked by the air layer, resulting in a sharp decrease in surface dose for the FFF mode as the gap increases.

This trend of dose reduction was clearly demonstrated in our quantitative analysis. The data revealed a highly linear negative correlation between the FFF/FF dose ratio (FNR) and air gap thickness, with a decay slope of approximately −2.1%/mm. This finding provides a more detailed characterization of dose variation patterns compared to previous studies on uniform slab gaps.^9^ Based on this linear model, a key clinical threshold of 2.32 mm was identified. This value carries both physical and clinical significance: when the gap between the bolus or immobilization device and the patient's skin is controlled within 2.32 mm (Zone I), the FFF mode, leveraging its softer spectrum, can effectively overcome the build‐up effect and provide superior target coverage compared to the FF mode; conversely, when the gap exceeds this threshold (Zone II), the FF mode demonstrates better dose robustness due to its harder spectrum and greater electron penetration capability. This threshold suggests that when creating treatment plans for areas where larger gaps are anticipated and difficult to eliminate (such as surgical scar depressions), the suitability of the FFF mode should be carefully evaluated.

It should be noted that this study deliberately employed a static single‐field geometric setup. This simplified physical model was designed to isolate the physical influence of spectral components on gap‐induced dose effects by eliminating the confounding variables introduced by multiple beam angles. In actual clinical practice, techniques such as volumetric modulated arc therapy (VMAT) or intensity‐modulated radio therapy (IMRT) involve beam incidence from multiple directions, and this multi‐directional dose contribution may produce a certain smoothing effect, potentially diluting the steep dose gradients observed in single‐field setups. Nevertheless, the fundamental physical principles revealed in this study likely remain applicable: the FFF spectrum is inherently more sensitive to low‐density media than the FF spectrum. Therefore, the 2.32 mm threshold derived from single‐field conditions can be considered a conservative reference, providing underlying physical rationale for beam selection in complex clinical scenarios.

Furthermore, in contrast to previous studies that predominantly employed uniform slab models,^18^, ^19^ a key innovation of this study is the introduction of the Type‐B gradient gap model. This model simulates clinically common non‐uniform fit conditions, such as those occurring at the lateral breast contour or neck curvature, and more realistically reflects the continuous variation of dose distribution with changing gap thickness. Through this gradient model, we not only captured the linear trend of dose attenuation but also observed complex behaviors of lateral electron transport at interfaces between non‐uniform media. The oblique incidence effect further compounded this complexity. The study showed that when oblique incidence and air gaps act together, the skin surface dose cold spots not only reach lower values but also shift downstream along the beam direction. This implies that when treating superficial targets with complex contours, reliance solely on TPS calculations may introduce deviations, with actual delivered doses often falling below algorithm predictions.

This study has certain limitations. First, the investigation was based on a static model and did not account for dynamic gap variations due to patient respiratory motion. In actual treatment, respiratory‐induced chest wall movement may cause periodic fluctuations in mean gap thickness, potentially leading to further averaging effects on cumulative dose. Second, while low‐density ABS was deliberately chosen for the support struts to minimize beam attenuation, their physical presence inevitably introduces minor dosimetric interference in the uniform gap models. Third, differences in linac head design among manufacturers (such as FF materials and geometry) may result in subtle variations in FF and FFF energy spectra, and the specific threshold values derived from the uRT‐linac 306c model in this study may require adjustment when generalized to other platforms. Future work will incorporate four‐dimensional computed tomography (4DCT) data to further investigate the combined effects of respiratory motion on skin dose under non‐uniform gap conditions.

## 5 CONCLUSION

This study systematically investigated the dosimetric impact of non‐uniform bolus fitting gaps on chest wall superficial radiotherapy using both FF and FFF modes. The presence of gradient air gaps on curved geometries severely disrupts CPE, leading to significant skin dose inhomogeneity and peripheral cold spots. Due to its softer energy spectrum and reduced secondary electron range, the FFF beam is markedly more sensitive to air gaps than the FF beam, exhibiting steeper dose fall‐offs at the gap interfaces. Furthermore, oblique beam incidence exacerbates these dosimetric degradations. Algorithmically, the standard CCC algorithm systematically underestimates the dose in complex low‐density gap regions, whereas the GPU‐accelerated MC algorithm demonstrates robust accuracy. Clinically, when air gaps exceed approximately 2.3 mm, it is highly recommended to either prioritize the FF beam mode or substantially optimize bolus conformality (e.g., using 3D‐printed customized bolus) to ensure adequate target coverage and minimize skin toxicity.

## AUTHOR CONTRIBUTIONS

**Long‐Gang Gui**: Conceptualization; methodology; investigation; formal analysis; writing—original draft. **Yin Poo**: Supervision; project administration; funding acquisition; writing—review & editing. All authors have read and agreed to the published version of the manuscript.

## CONFLICT OF INTEREST STATEMENT

The authors declare no conflicts of interests.

## Data Availability

The data that support the findings of this study are available from the corresponding author upon reasonable request.
